# Supplementary material for: Rapid Estimation of Soil Pb Concentration Based on Spectral Feature Screening and Multi-Strategy Spectral Fusion
Source: Sensors (Basel). 2023 Sep 6;23(18):7707. doi: 10.3390/s23187707 (PMC10538168; doi:10.3390/s23187707)
Supplement: Supplementary file 1 [file sensors-23-07707-s001.zip › Table S1 and S2.pdf]

Supplementary Materials:

Table S1. Descriptive statistics of characterizations information of soil samples. Unit: g/kg

Characterizations	Minimum	Maximum	Mean	Standard deviation	Median	CV
N1	7.68	3367	58.27	226.72	39.54	389.06%
N2	4.5	551	88.46	65.98	72.76	74.59%
K	58.9	1206	390.37	204.55	363.5	52.40%
P	4.37	665.4	89.01	94.2	58.82	105.83%
Organic carbon	5.51	18.72	13.91	86.94	6.47	95.24%

Table S2. Information on particle size composition of soil samples

	clay(＜2μm)	silt(2-50μm)	sand(50-2000μm)
Percentage	16.93%	77.38%	5.69%
