# Supplementary figures and images for: Rapid Estimation of Soil Pb Concentration Based on Spectral Feature Screening and Multi-Strategy Spectral Fusion
Source: Sensors (Basel). 2023 Sep 6;23(18):7707. doi: 10.3390/s23187707 (PMC10538168; doi:10.3390/s23187707)

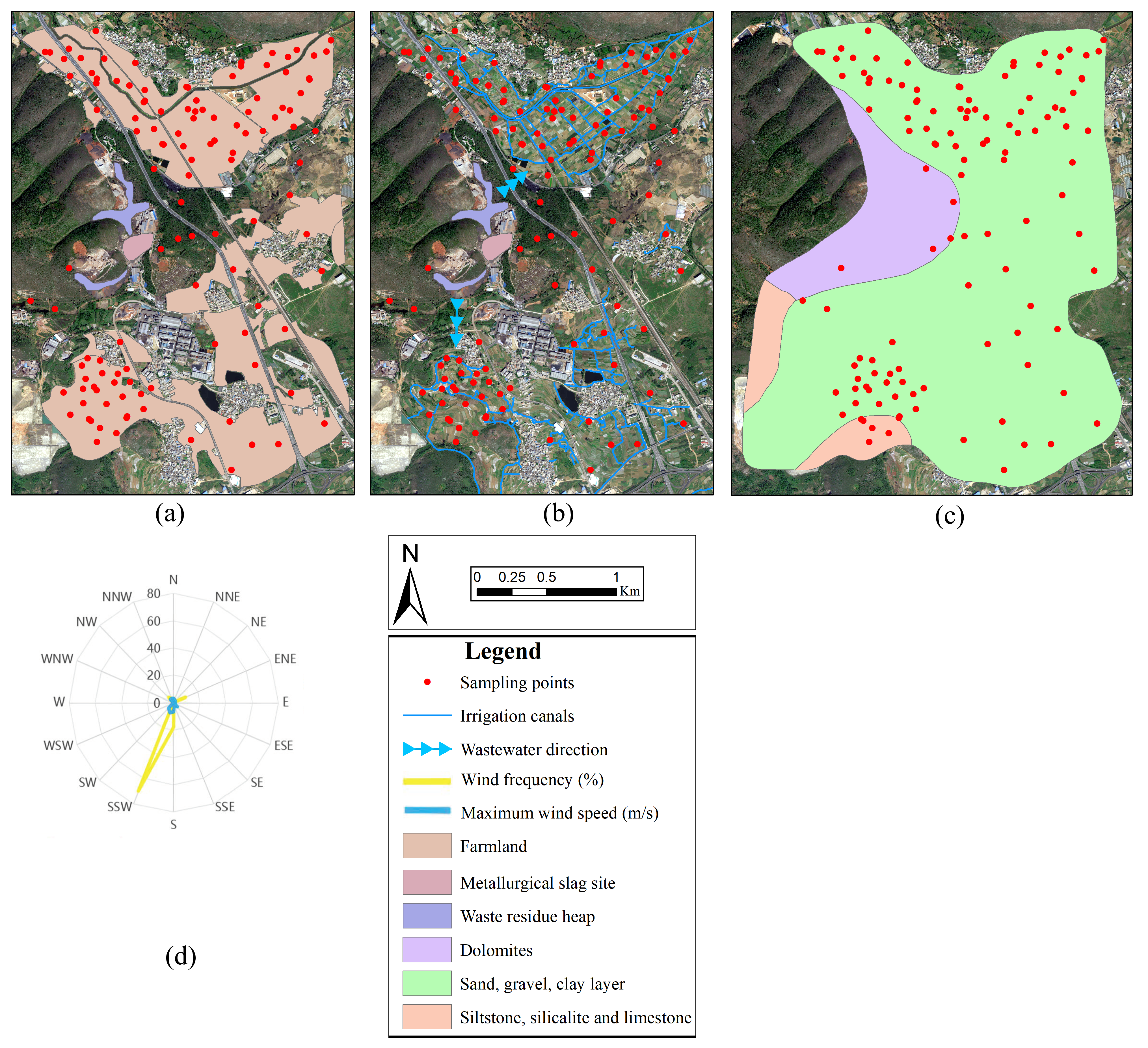

Supplement: Supplementary file 1 [file sensors-23-07707-s001.zip › Figure S1. Supplementary infographic of the study area.tif]

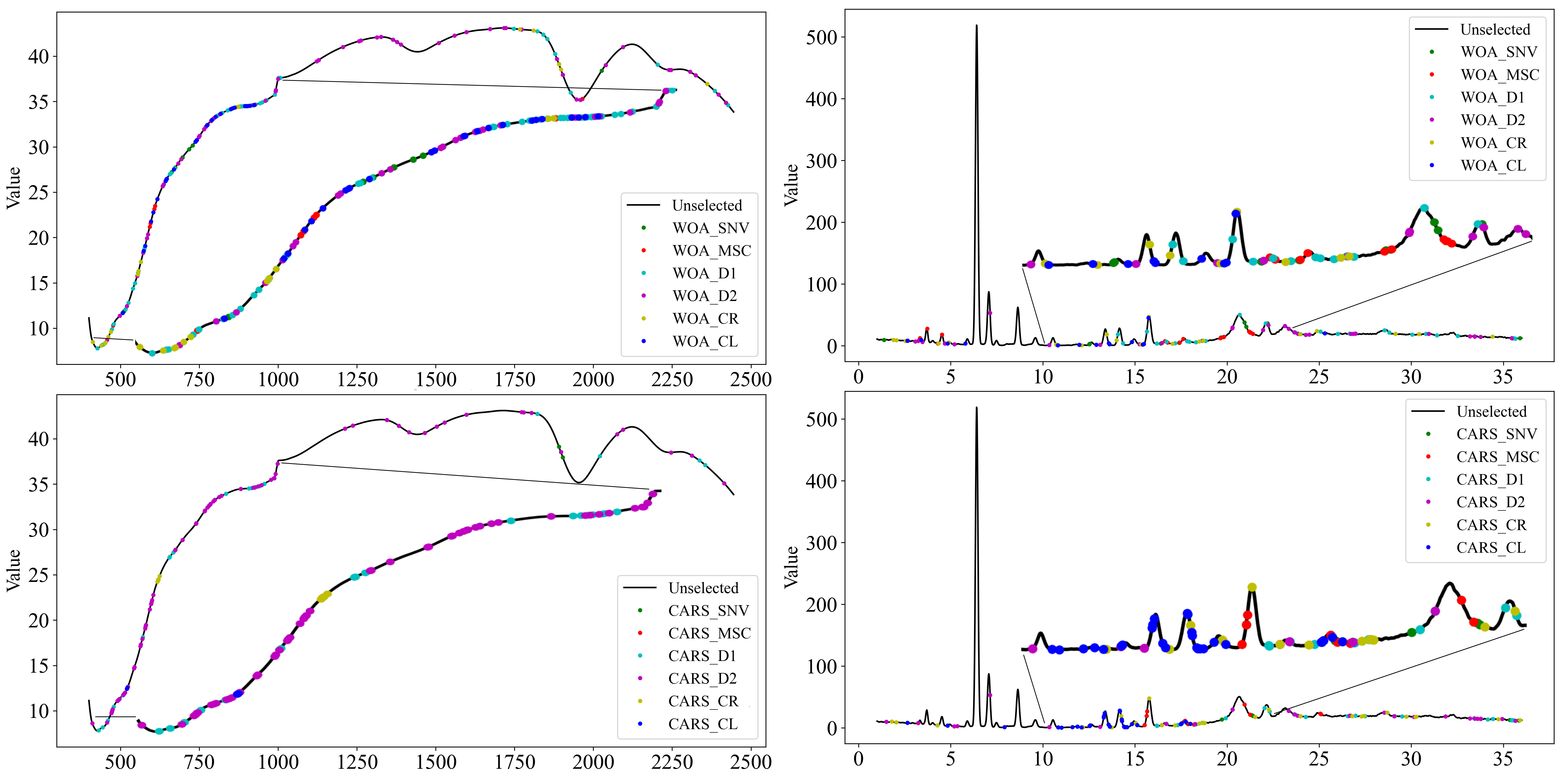

Supplement: Supplementary file 1 [file sensors-23-07707-s001.zip › Figure S10. Feature spectrum position chart.tif]

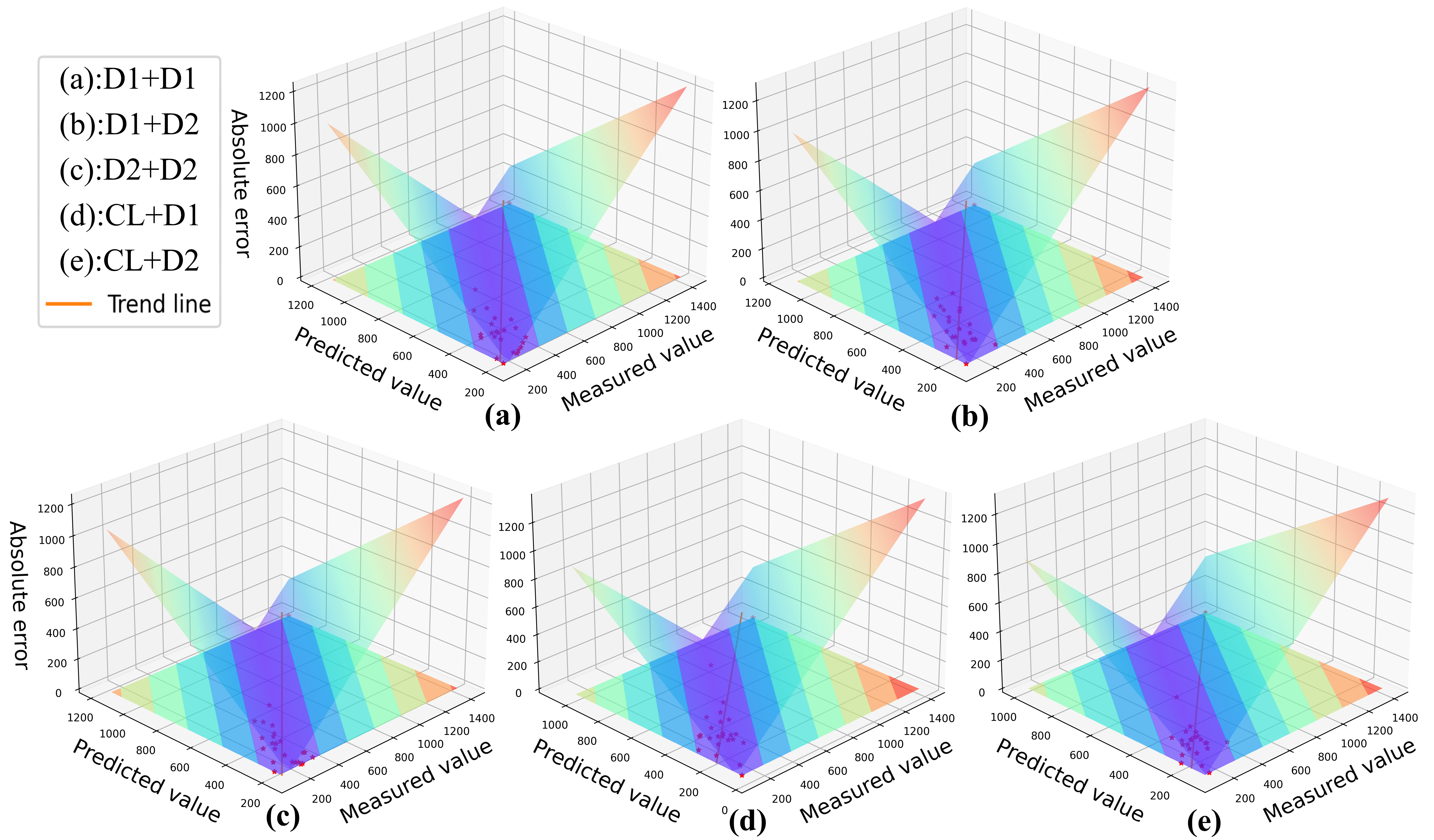

Supplement: Supplementary file 1 [file sensors-23-07707-s001.zip › Figure S11. The scatter plot of the measured and estimated values of the soil Pb content estimation model constructed by the WOA algorithm.tif]

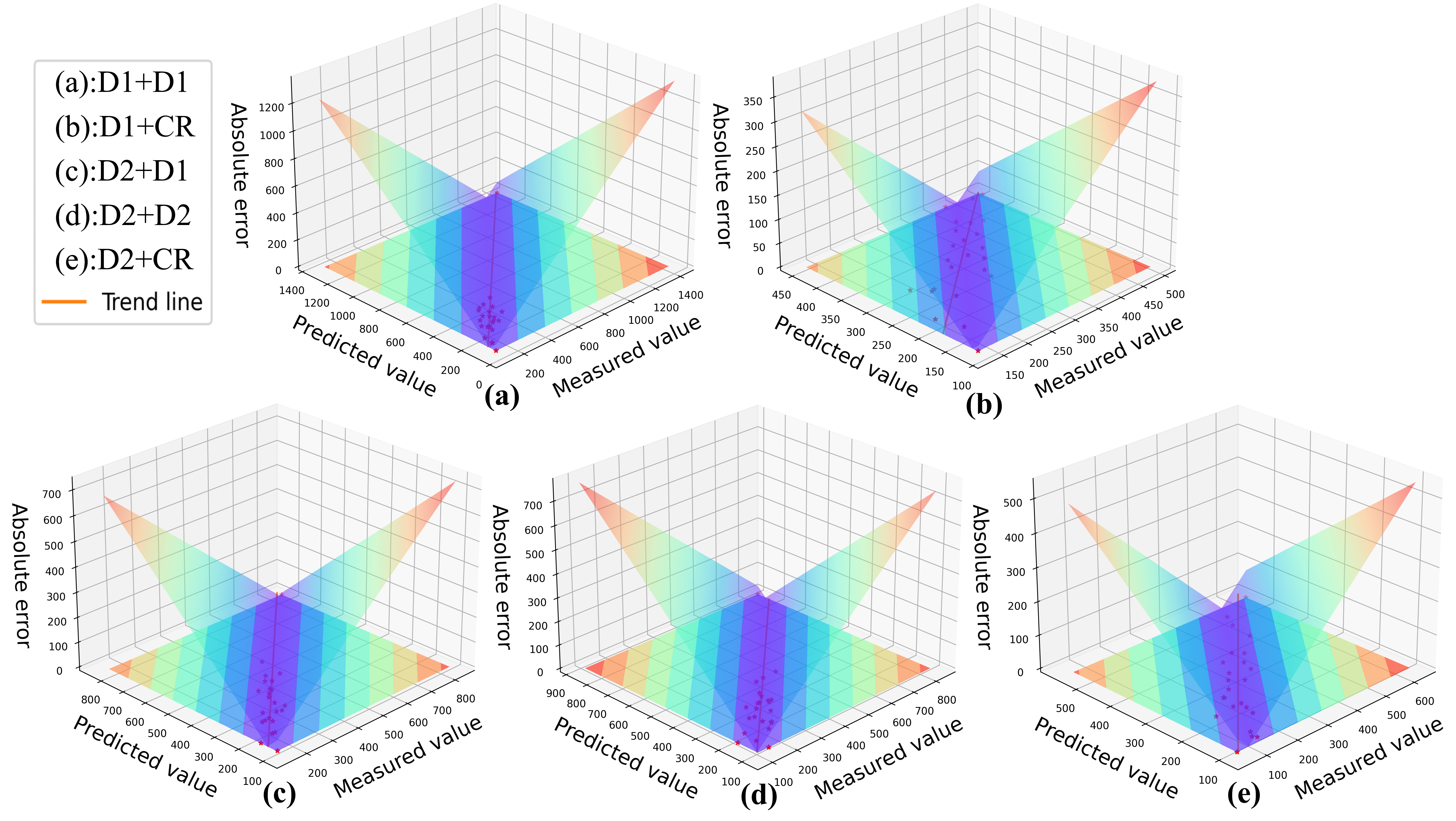

Supplement: Supplementary file 1 [file sensors-23-07707-s001.zip › Figure S12. The scatter plot of the measured and estimated values of the soil Pb content estimation model built by the CARS algorithm.tif]

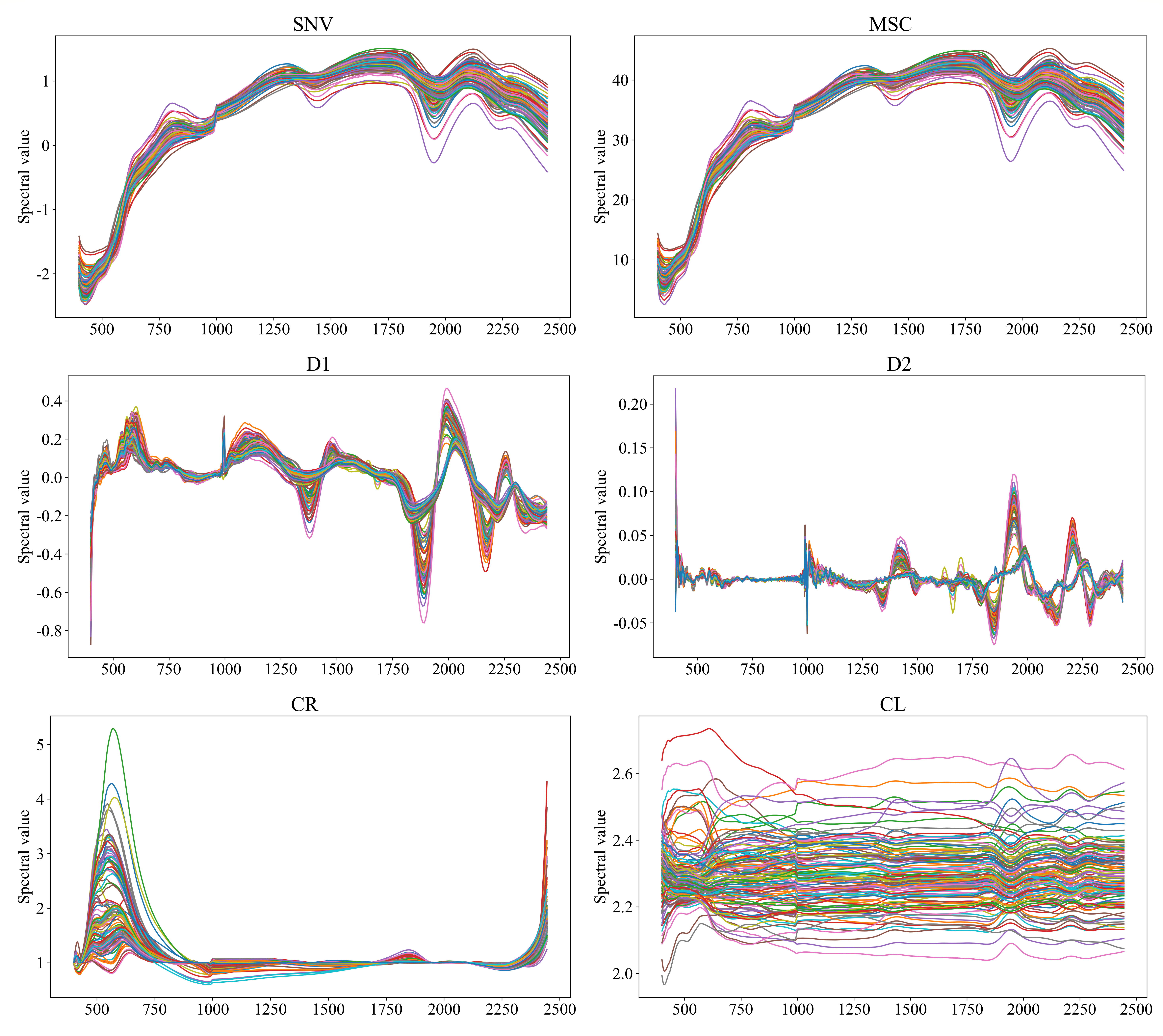

Supplement: Supplementary file 1 [file sensors-23-07707-s001.zip › Figure S2. Spectral transformation of vis-NIR.tif]

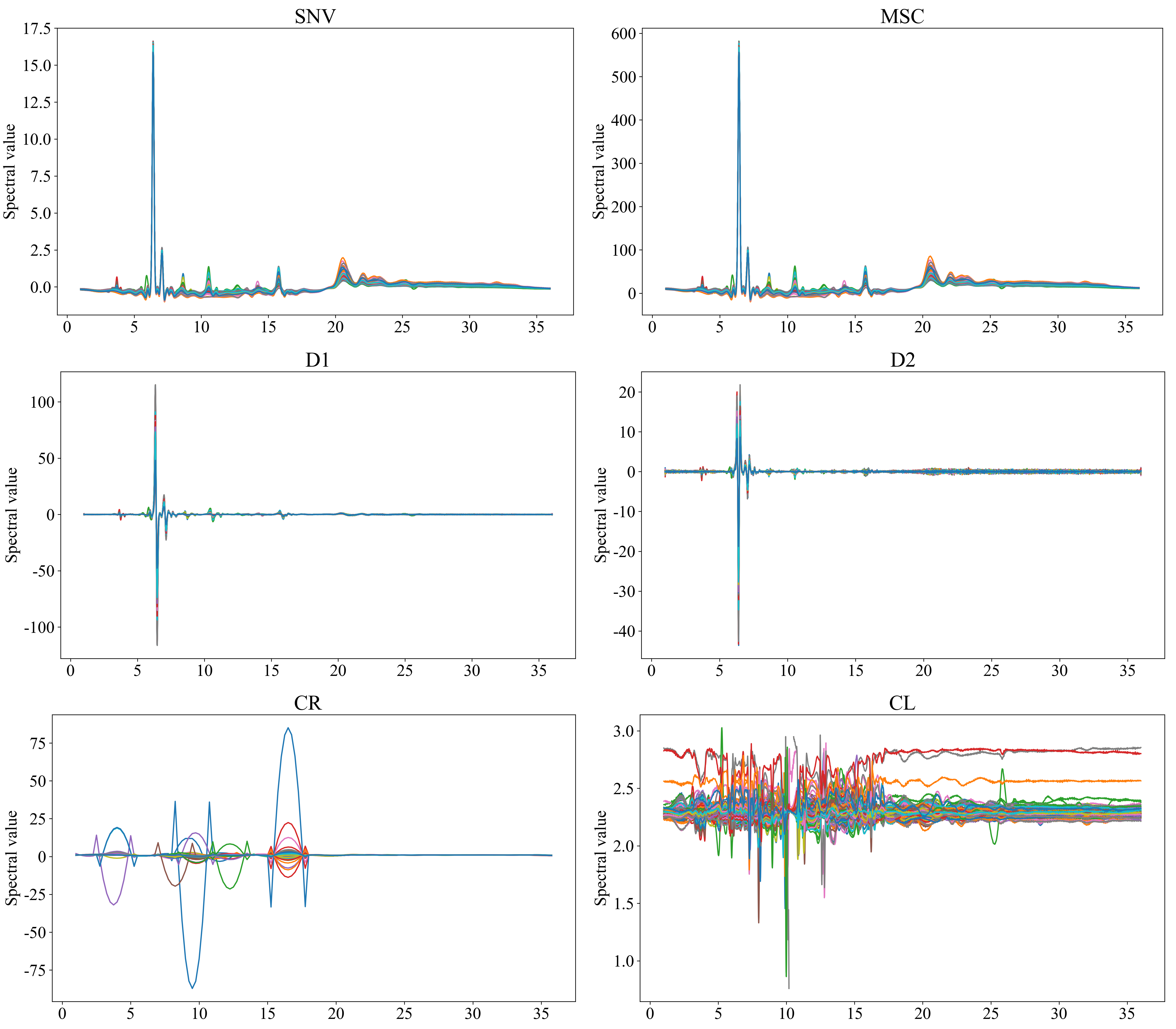

Supplement: Supplementary file 1 [file sensors-23-07707-s001.zip › Figure S3. Spectral transformation of XRF.tif]

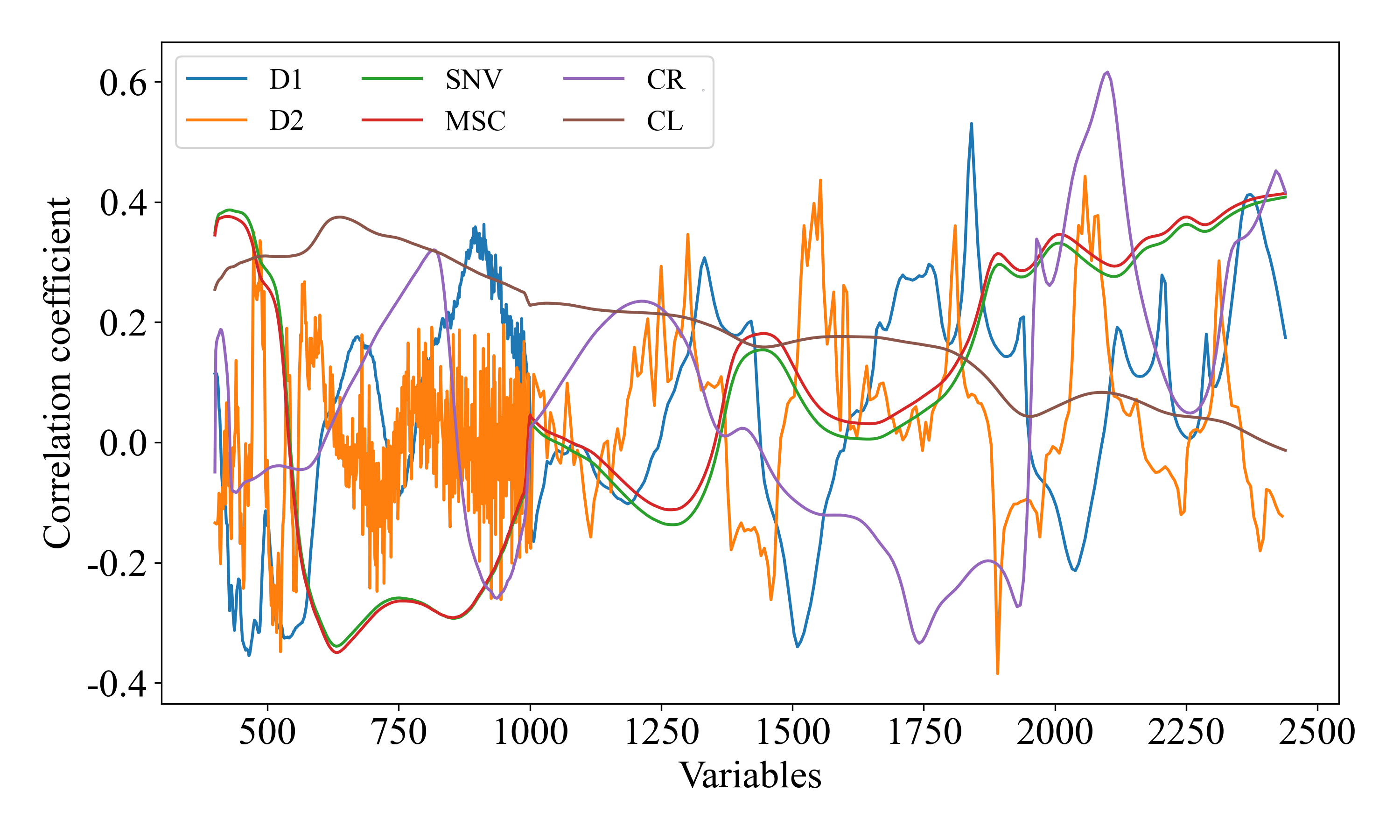

Supplement: Supplementary file 1 [file sensors-23-07707-s001.zip › Figure S4. PCC of vis-NIR.tif]

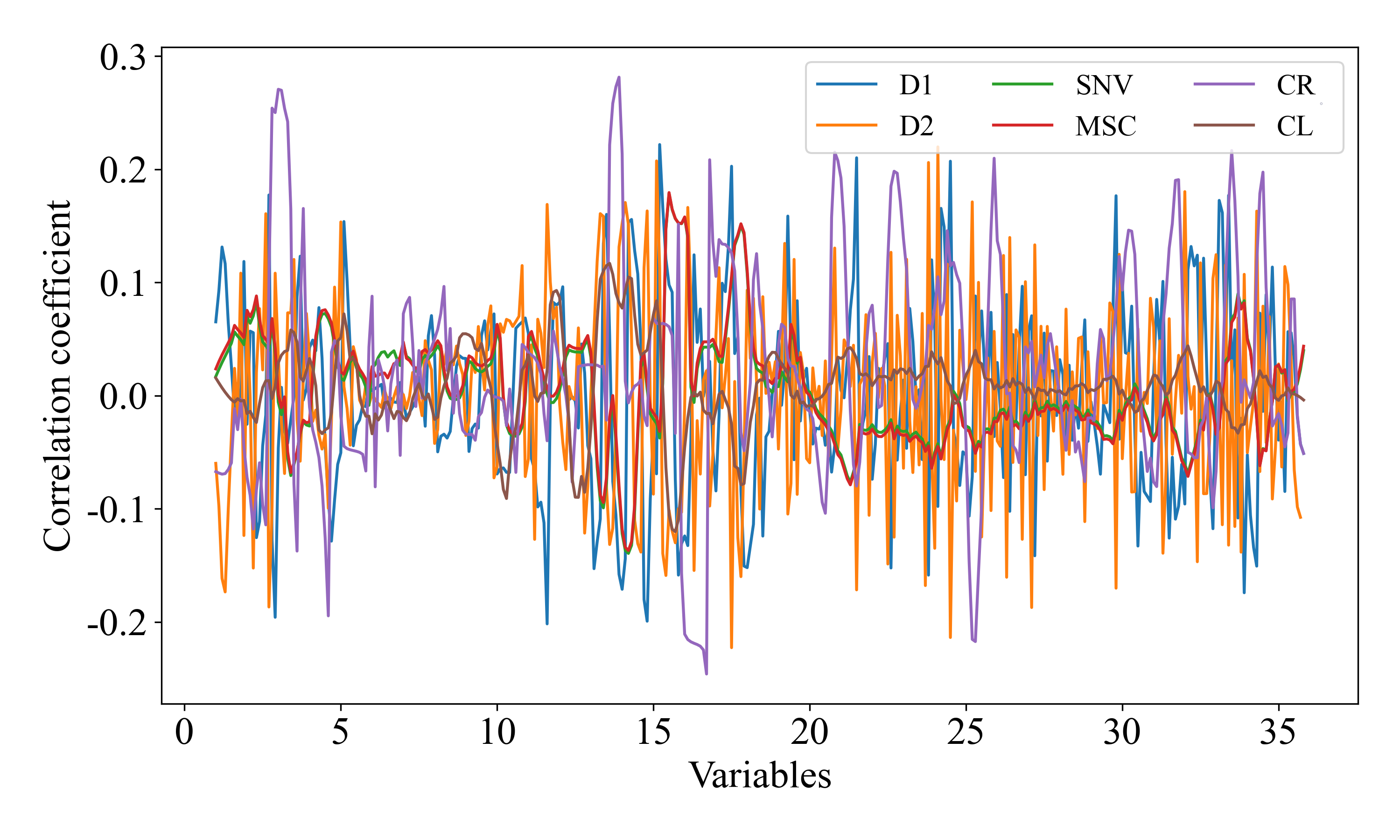

Supplement: Supplementary file 1 [file sensors-23-07707-s001.zip › Figure S5. PCC of XRF.tif]

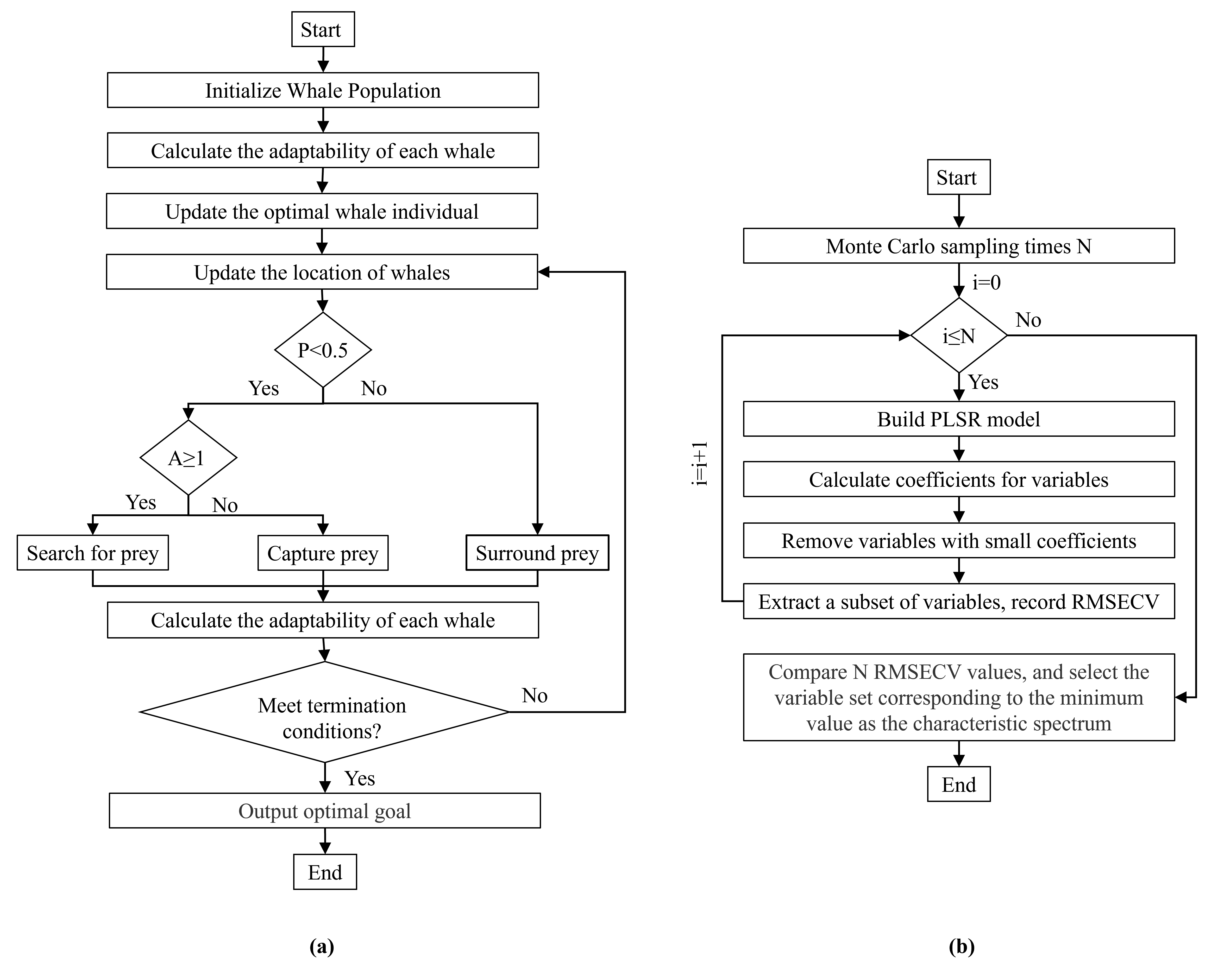

Supplement: Supplementary file 1 [file sensors-23-07707-s001.zip › Figure S6. Flowcharts of the WOA and CARS.tif]

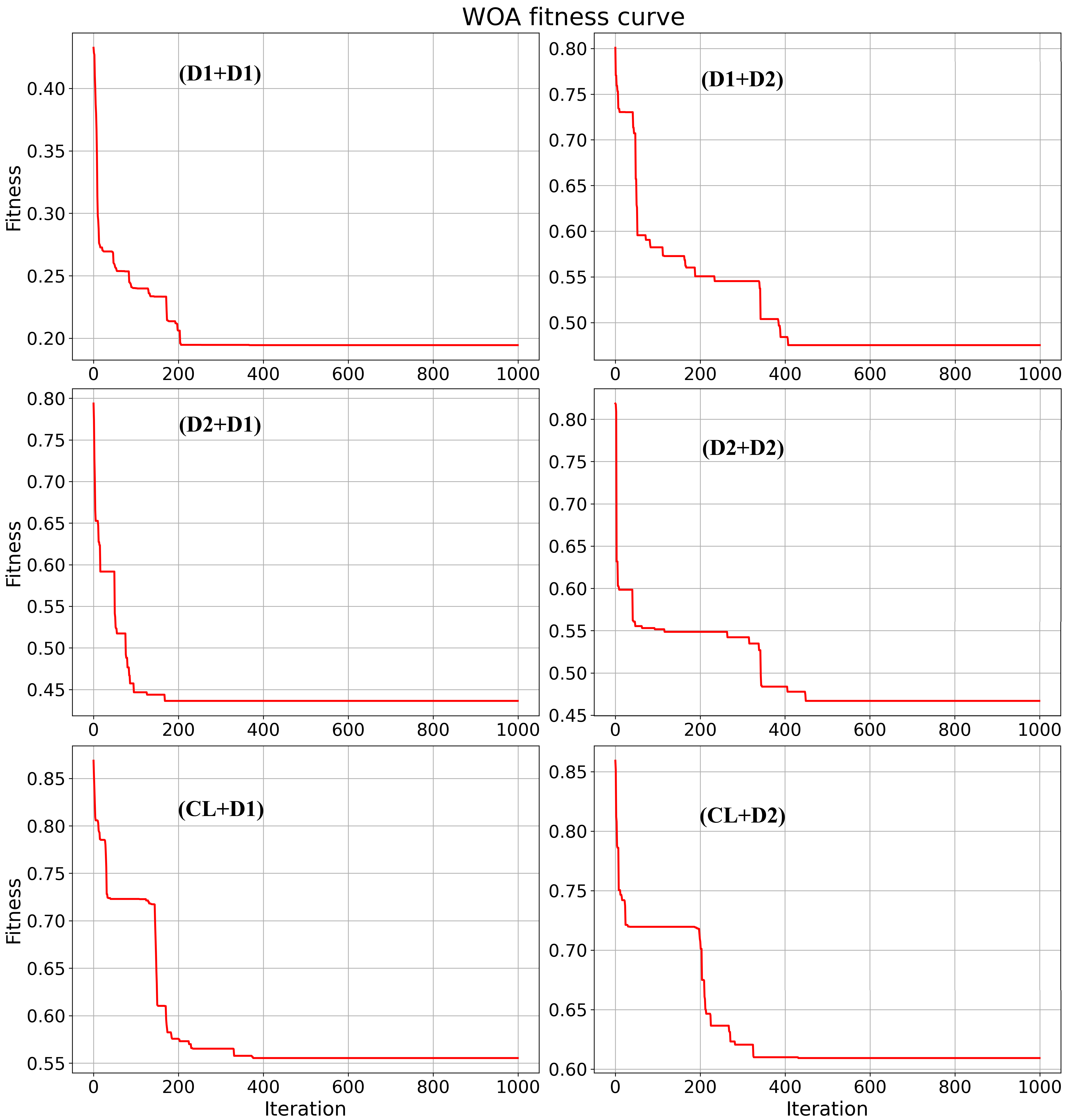

Supplement: Supplementary file 1 [file sensors-23-07707-s001.zip › Figure S7. Fitness curve of WOA.tif]

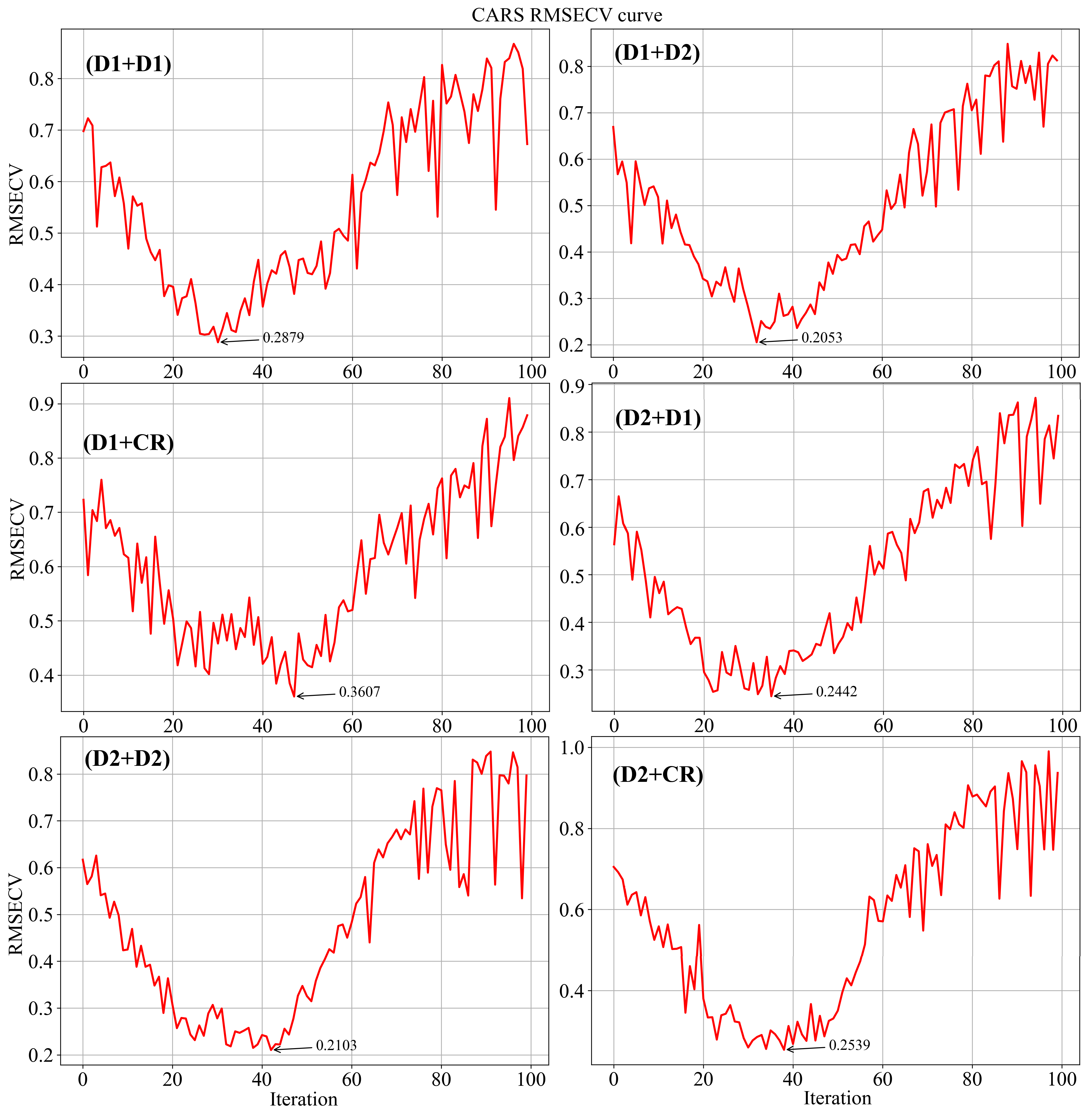

Supplement: Supplementary file 1 [file sensors-23-07707-s001.zip › Figure S8. RMSECV curve of CARS.tif]

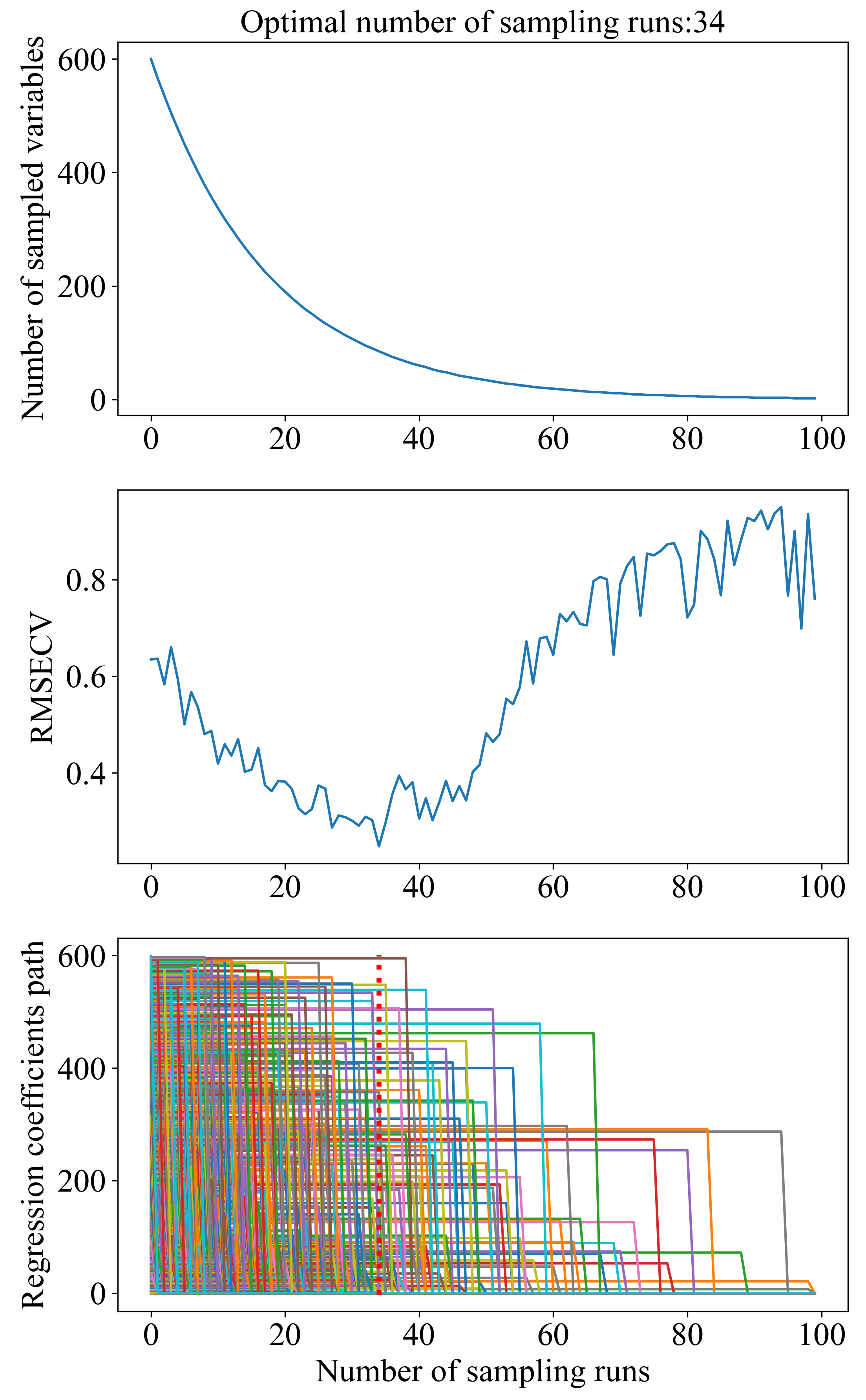

Supplement: Supplementary file 1 [file sensors-23-07707-s001.zip › Figure S9. Iteration curve of CARS.tif]
